# Leveraging Professional Sports Teams to Encourage Healthy Behavior: A Review of 4 Years of Calgary Flames Health Training Camp Events

**DOI:** 10.3389/fpubh.2020.553434

**Published:** 2020-11-20

**Authors:** Elaine M. Ori, Tanya R. Berry, Gavin R. McCormack, Kelly R. Brett, George A. Lambros, William A. Ghali

**Affiliations:** ^1^Faculty of Health, Community and Education, Mount Royal University, Calgary, AB, Canada; ^2^Faculty of Kinesiology, Recreation and Sport, University of Alberta, Edmonton, AB, Canada; ^3^Cumming School of Medicine, University of Calgary, Calgary, AB, Canada; ^4^O'Brien Institute for Public Health, University of Calgary, Calgary, AB, Canada; ^5^Innovative Sport Medicine, Calgary, AB, Canada; ^6^Canadian Pacific Railway, Calgary, AB, Canada

**Keywords:** professional sporting organizations, healthy communities, community health promotion, healthy cities, health behavior

## Abstract

Professional sporting teams may be well-positioned to act as promoters of health behaviors given their fixture within a community, and association with physical activity, nutrition, and other healthy behaviors. Over 4 years, the Calgary Flames Sport and Entertainment Corporation in conjunction with local health promotion professionals, delivered a health promotion event to the public, The Calgary Flames Health Training Camp (FHTC) in Calgary, Alberta, Canada. The purpose of these annual events has been to inspire and encourage healthy behavior uptake and adherence. A description of the FHTC over each of 4 years (2015–2018), lessons learned, and some evaluative work done alongside the event on 2 of the 4 years. In 2017, self-report surveys were administered to event attendees to assess current health status including physical activity, socio-cognitive variables, health information preference, and intention to make healthful behavior change based on event attendance. Biometric data was collected including blood pressure, height, weight, and resting heart rate. Evaluations of the four consecutive events showed that the Calgary Flames Sport and Entertainment Corporation has an ability to attract substantial numbers of the general public to attend FHTC events. Self-report measures from 2017 suggest that already-active populations may be most interested in attending however, the events do appear to inspire attendees to consider behavioral changes for health. The events helped to identify individuals with health risks requiring medical attention but has not yet resulted in known behavior changes. Positive community health impacts may arise from collaboration between health promoters and professional sporting organizations.

## Introduction

When the Chicago Cubs won the World Series of Baseball in 2016 after a 108 year championship drought, the ensuing World Series Champions Parade in Chicago drew an estimated 5 million people (1). Reports indicate that the Cubs' Champions parade ranks in the top 10 largest gatherings in human history, and the largest ever recorded in the Western hemisphere (2). Sporting events have a demonstrated ability to draw crowds of people together around a shared interest. Even the Olympics and FIFA World Cup of Soccer consistently draw millions of spectators, with 8.2 million attending the 2012 London Games and 3.4 million attending the 2014 FIFA World Cup (3). Indeed, sport has the power to unite and gather regardless of age, background, or language. Given this ability to draw crowds from a multiplicity of backgrounds, sporting events, and sports franchises may provide an opportunity to leverage professional sports organizations in the interest of improving communities. In this article, we review the potential contributions that professional sport franchises can make to the health of their communities including a description of four successive annual health promotion events staged by the Calgary Flames, a professional hockey team in Calgary, Alberta, Canada. The information presented may be of relevance to similar organizations considering such events, and of considerable relevance to both the sociological and psychological study of public health including factors that motivate and inspire populations toward health and well-being.

### The Potential Influence of Sports Franchises

Extant research suggests sports teams are well-poised to convey health promoting messages and activities, contributing to improved health outcomes of community members (4–7). Sporting organizations and franchises are uniquely poised to draw individuals from across broad geographic regions as well as extending the reach to those from varying social backgrounds (4, 8). Much of the current work in this area has focused on drawing from crowds already attending sporting events. For example, team initiatives have centered around creating healthy environments for fans attending games, such as healthful nutrition options at concessions, tobacco-free stadia, responsible alcohol consumption, active breaks (i.e., follow-along exercises, dancing) between game plays, green commuting to/from games, health screening programs, and promotion of healthy behaviors (4, 6–9). The relative success of these initiatives has been seen by community stakeholders, health partners, teams, and players reporting favorable feedback and increased participation in promoted health behaviors while attending games (4, 6, 9). Physical activity (PA) promotion by sporting teams has also been well-established, including initiatives to engage children, youth, and local community (7, 9, 10). It is not known however, if these healthy behaviors extend beyond game attendance, nor if there is any significant community-wide long-term effects resulting from these initiatives.

In an attempt to increase favorable health behaviors of their fan bases, professional sporting organizations have taken several approaches to health promotion. The EuroFIT program aims to leverage football fan loyalty in 15 European professional football clubs through enrolling overweight and obese adult men aged 35–65 years, into intervention programs targeting PA and nutrition, based on a previous iteration of the program, Football Fans in Training (11, 12). This 12 week, enrolled, targeted intervention includes behavior change classroom work combined with coached PA sessions taking place at the football club's stadium (11). This intervention has also been applied to Canadian Junior A and Ontario Hockey League teams (13, 14). However, the approach is limiting to individuals that “fit the profile” of the intervention, thus eliminating a large component of a team's fan base such as youth, young adults, women, and older adults. Further, given the programmatic nature of the intervention, its impact on local communities is limited and thus does not fully leverage a sporting team's potential reach.

Currently more than one quarter of adults (1.4 billion) worldwide are insufficiently active to achieve optimal health benefits (15). The number of adults not attaining the recommended guidelines of 150 min of moderate-vigorous PA per week is increasing (16). Among global populations of all ages, nutrition challenges also remain problematic with fruit and vegetable consumption below recommended nutritional guidelines of ≥5 fruits and vegetables daily (17–19). Additionally, 39% of adults 18 years of age and older are considered clinically overweight, and as many as 13% are living with obesity, a significant contributor to all-cause mortality and chronic disease (20). Given these trends, it is of interest for health promoters and professionals to identify ways in which to encourage healthy behaviors to improve the health of all populations.

Community-based health promotion events have shown that the use of health screening, counseling services, clinics, and health education are effective tools for promoting health behaviors and awareness (21–23). Recent studies have shown that health promotion events have been successful at increasing awareness of healthy lifestyle opportunities, identifying health risk factors, and assessing readiness to change (22, 24). However, health behaviors and health risk factors may already be known to the public, due to long-term promotion by public health campaigns (22). Although previous research has evaluated health promotion events for reach and effectiveness, there remains limited evidence examining the effects of health promoting events on a general public (21, 25). Given the need for improvement in the uptake of healthy behaviors, one approach may be to leverage the brand of professional, local sporting teams to promote and encourage community-wide uptake and adherence to PA and sport, improved nutrition, and overall healthier lifestyle. Local sporting teams are permanent fixtures within communities, exercising broad reach and visibility within their geographic regions and are poised with a unique opportunity to encourage healthy lifestyles over a prolonged period of time.

## Context

Over a 4 year period (2015–2018), the Calgary Flames Sport and Entertainment Corporation (herein known as “The Corporation”) hosted a series of health promotion events. Using a multi-agency approach, The Corporation first launched the Calgary Flames Health Training Camp (FHTC) event in 2015 (Calgary, Alberta, Canada), as a 1 day health promotion event centered on increasing PA, healthy nutrition practices, and health screening within the community. Since that inaugural event in 2015, the FHTC has been held annually with a slightly varying format each year. Overall aims of the annual events have been to inspire the local community to lead healthier lifestyles. Health care providers, health promotion practitioners, and trainees associated with local universities participated in event planning and service delivery. The Corporation provided funding for the 1 day event, inviting any and all members of the public with an event promotion tag line “*Come with a goal, leave with a plan,”* encouraging the local community to attend the event as means to learn more about healthy lifestyles, and to meet with health professionals capable of providing healthy lifestyle advice.

### Setting

The City of Calgary is a large, Canadian city in the Western province of Alberta with a population of ~1.23 million residents (26). With a mean age of 37.4 years, Calgary has the lowest average age among major Canadian cities; 70.2% of the Calgary population is between 15–64 years (27). The Calgary Flames Sport and Entertainment Corporation (herein known as “The Corporation”) is the owner of four local professional sporting teams that operate within the City of Calgary (Calgary Flames, National Hockey League; Calgary Roughnecks, National Lacrosse League; Calgary Hitmen major junior Western Hockey League; Calgary Stampeders, Canadian Football League).

## Detail

### Formative Process

Beginning in October, 2014, The Corporation convened members of senior management, medical staff, event staff, and local health promotion and public health researchers and professionals, to establish a multidisciplinary team that would assess the viability of a local public health event. This group of individuals would form the Steering Committee for the duration of all FHTC events. The framework for the event would be informed by the Steering Committee, executed by staff and volunteers of The Corporation, and funded by The Corporation, using The Corporation's visibility within the community to promote the event, an early stage format of community-based action research (7). Thus, varying perspectives on intervention approaches and health promotion efforts informed discussions and an inaugural event was planned to pilot the event.

Given the varied nature of the multidisciplinary team, several foci were established for assessing health metrics at the event, as well as practices to promote health behaviors to attendees at the event. Public health professionals recommended that the event should include health care teams comprised of physicians and student nurses to provide personal health assessments for event attendees such as health screen questionnaires (28), heart rate, blood pressure, and anthropometric measurements. This approach was based on Canadian national data suggesting an increasing trend in diagnoses of Diabetes Mellitus and Hypertension (29). Attendees would then be able to review their results with nurse/physician teams at the event, in a private counseling session, allowing for identification of at-risk individuals for referral to a primary care provider for future follow-up. To address the concerns of physical activity promoters looking to improve low-levels of physical activity (30), it was determined that attendees would be able to meet with a personal trainer on site, perform field-based fitness testing (31), and receive individualized exercise advice from exercise physiologists and personal trainers. Collectively, health assessments and exercise testing/prescription would be available at the event as a means of determining a baseline, general health indicator (see Table 1 for all activities offered).

**Table 1:** Activities offered at the Calgary Flames annual health training camp events.

**Activity**	**Description**	**Offered**			
		**2015**	**2016**	**2017**	**2018**
Health screening with nurse/physician	One-on-one meeting for blood pressure checks, hip: waist circumference measurements, height, weight, and health history via CanRisk Questionnaire, PA Readiness Questionnaire; private review of results	x	x	x	x
Fitness testing	Submaximal fitness test: modified Canadian Aerobic Fitness Test	x			
Personal training consultation	Individualized exercise advice/prescription		x	x	x
Group fitness classes	20 min schedule of classes including: resistance training, cycle, and yoga classes, all taught by certified group fitness instructors		x	x	
Children's fitness activities	Group obstacle course races, encouraging physical literacy skills such as crawling, skipping, jumping, running, and throwing		x	x	
Children's fun fitness assessments	Group-based modified beep test, timed sit-up, and push-up tests	x			
Family open skate	Attendees used the Olympic training ice for free-skate		x	x	
Try-sport booths	Local agencies with equipment/space set up for trying a new sport, information for recreational sport registration		x	x	
Health agency booths	Regional health authority providing free enrollment into chronic disease management programs; YMCA provided membership information and enrollment				x
Open kitchen/cooking demonstrations	The Calgary Flames Catering Services hosted an open-kitchen demonstrating healthy cooking techniques, meal planning, and prepared food samples	x	x	x	
Nutrition booth & food guide presentations	Students from a local college nutrition program hosted a booth providing information on Canada's Food Guide, healthy nutrition behaviors and healthy recipes	x	x		x
Speaker series	Health-based lectures from local experts (professors, local celebrities) about various health topics such as healthy behavior change	x	x	x	
Swag bag	FHTC-branded hats, shirts, and hockey pucks for all attendees	x	x	x	
Free admission to Canadian sports hall of fame	Attendees could visit the museum at no cost		x	x	
Player meet & greet/autographs	Attendees could meet and receive autographs from current and alumni players	x	x	x	x

*x = indicates this activity was offered*.

It was anticipated that the use of The Corporation's existing communication channels (social media, direct email, television, radio) would be sufficient for promoting the event to the Calgary public. High attendance and event effectiveness were anticipated given the novelty of the event in the Calgary area, and The Corporation's ability to attract a broad and diverse following for each of their professional teams. Further, the variety of activities offered at the event were seen as a potential influence on both attendance and health promotion as strategies were incorporated to address perceived barriers to health behavior uptake (e.g., on-site registration for sport/recreation clubs), improve healthy behavior knowledge (e.g., healthy cooking techniques and recipes), and provide resources to individuals considered at-risk for poor health (e.g., referral to physician network).

### Flames Health Training Camp 2015

In January 2015, the first annual FHTC was hosted at the Saddledome (Calgary), the home arena for three of the five sports teams owned by The Corporation (Calgary Flames, National Hockey League; Calgary Roughnecks, National Lacrosse League; Calgary Hitmen major junior Western Hockey League). Early January was selected so as to coincide with New Year's resolutions. To anticipate attendance rates, interested participants registered online, free of charge. In addition to health assessments and exercise testing/prescription, children and youth were also encouraged to attend the event, and were provided several recreation-based fitness opportunities in which to participate. Children 8–12 years were given the opportunity to participate in fitness assessments and friendly competition with attending professional athletes, were encouraged to participate for fun and were not evaluated against any standardized criteria.

Attendees may have also taken part in other non-personalized activities at the event, or participating in group or family fitness activities. Event attendance was tabulated by organizers with approximately 700 individuals attending. As the 2015 event served as a pilot event, no formal evaluations were undertaken. It was noted however, that the 2015 FHTC took place during Esso Minor Hockey Week, a highly attended annual, week-long hockey tournament in Calgary involving minor hockey players of all ages, which may have impacted 2015 FHTC event attendance.

### Flames Health Training Camp 2016

In March 2016, the second iteration of the FHTC was offered in Calgary anticipating that this timing might increase event attendance from 2015, and to decrease time conflicts with other sporting events taking place in Calgary. In addition to partnerships with two local universities, the 2016 event was co-hosted by WinSport at Calgary Olympic Park. At the time of the 2016 FHTC, WinSport was the site of the Olympic athlete development center, a high performance training center for Canadian Olympic athletes to train and work. Co-branding of both WinSport and The Corporation as well as local Olympians was used to promote and inspire the power of sport for PA within the Calgary community.

Similar to 2015, an array of activities and health-promoting opportunities were available to attendees with additional leisure activities on-site (see Table 1). A total of 96 individuals attended one-on-one health assessments with nurses/physicians. In lieu of fitness tests, prescriptive exercise from personal fitness trainers was offered to interested participants. Attendees were able to pre-register online or drop-in. Youth and children in attendance were able to join, and were hosted by personal fitness trainers and professional/Olympic athletes, centering activities on fun and enjoyment, and on participation in various activities. Again event attendance was tabulated by organizers; ~2,000 individuals attended the 2016 event. No formal evaluation of the 2016 FHTC beyond the inventory of elements and activities just provided.

### Flames Health Training Camp 2017

The Annual FHTC was again hosted in March in 2017, and co-hosted with WinSport at Calgary Olympic Park. No changes were made from the 2016 event, though ethics approval was sought from the University of Calgary Conjoint Health Research Ethics Board for a self-report, cross-sectional examination of 2017 adult attendees. This was the 1st year a formal evaluation of the FHTC was undertaken.

#### Methods

Participants completed a waiver of informed consent then were given paper-and-pen surveys asking a series of lifestyle and event-related questions, including participation in PA, reasons for attending the FHTC, and intentions to make lifestyle changes for improved health after attending the FHTC. Respondents completed a self-reported PA questionnaire (32) capturing how many times per week they engage in PA, and for the average duration of that activity, in minutes. Participants completing the survey were entered for a chance to win a Calgary Flames prize pack consisting of a Calgary Flames Hockey jersey or game tickets.

#### Results

From an estimated 700 attendees, 54 adult respondents agreed to complete the on-site survey and ranged in age from 20 through 67 years (*M* = 41.26, *SD* = 10.50). Respondents had a mean body mass index (BMI) of 27.74 kg/m^2^; (*n* = 52), indicating that overall survey respondents were classified as overweight (range: 25–29.9 kg/m^2^) but not obese (33). Overall mean blood pressure (BP) was reported as 117/74 mmHg, indicating that respondents tested overall had blood pressures within the optimal healthy range: ≤120/80 mmHg (34). Survey respondents reported accumulating on average 214 min of total moderate-to-vigorous PA per week. This suggests that in 2017, FHTC attendees completing the questionnaire were a generally active subset of the general population, demonstrating PA well above the minimum guidelines for achieving health benefits (16). Approximately one-third of respondents (35.19%) reported their primary reason for attending the event was to learn how they could improve their health. Approximately two-fifths (40.70%) of respondents reported the station that provide information about actions that could be taken to improve health as their most preferred station at the event. Notably, 65% of respondents reported that their participation at the 2017 FHTC motivated them to modify an unhealthy behavior. A full list of survey results are reported in Table 2.

**Table 2:** 2017 FHTC survey data.

		**N**	**%**	**M**	**SD**
**Age**					
	Male	30	60.00	40.47	11.65
	Female	20	40.00	41.35	9.57
Body mass index (kg/m^2^)		51	94.44	27.74	4.94
Blood pressure		11	20.37	117/75	-
**Highest level of education**					
	High school diploma	9	18.75	-	-
	College certificate/diploma	16	33.33	-	-
	Bachelor's degree	16	33.33	-	-
	Master's degree	3	6.25	-	-
	Doctoral degree	2	4.17	-	-
	Professional degree	2	4.17	-	-
**Smoking**					
	Regular smoker	1	2.04	-	-
	Social smoker	2	4.08	-	-
	Non-smoker	46	93.88	-	-
**Physical activity participation**					
	Strenuous–times per week	50	92.59	3.76	3.95
	Strenuous–minutes per time	47	87.03	57.04	54.64
	Moderate–times per week	44	81.48	3.32	2.12
	Moderate–minutes per time	41	75.93	63.98	92.33
	Mild physical–per week	44	81.48	3.77	4.71
	Mild–minutes per time	36	66.67	94.86	158.96
**Preferred FHTC stations**					
	Learning about the effects of high blood pressure/overweight/ obesity/high cholesterol/high blood glucose/smoking on my health	4	7.40	-	-
	Learning what actions I can take to improve my health	22	40.70		
	Talking to health care professionals in a relaxed setting	8	14.80	-	-
	Talking to more than one kind of health professional (i.e., physician, nurse, personal trainer)	9	16.70	-	-
	Keynote speakers	5	9.26	-	-
	Healthy cooking and nutrition	20	37.04	-	-
	Children's activities	20	37.04	-	-
	Open skate	8	14.81	-	-
**Reasons for attending**				-	-
	Free health screenings	13	24.07	-	-
	Wanting to learn more about healthy living and disease prevention	19	35.19	-	-
	Relatives/friends attending the event	11	20.37	-	-
	Meet and greet with athletes	16	29.63	-	-
	Free incentives (i.e., personal training, Flames memorabilia, etc.)	18	33.33	-	-

### Flames Health Training Camp 2018

To meet the objectives of The Corporation which included making Calgary the “*healthiest city in the world,”* steering committee members agreed to take the FHTC initiative to the community. This was determined as the location of WinSport (site of the 2016 and 2017 events) was not easily accessible for all Calgarians (e.g., lack of easy public transportation), making access to the event limiting to those living in regions of the city distant from WinSport. Further, it was determined that the WinSport facility, a performance training center, host to Olympic and future Olympic athlete development training, and part of the Calgary Olympic Park campus, may have been limiting to novice exercisers and those interested in learning more but who otherwise did not consider themselves athletes. Further, results of the 2017 survey indicated that free health screenings, and opportunity to learn about health improvement were among the primary reasons for attending the event. The steering committee determined that scaling back the event would allow for a more focused, health-driven approach. Therefore, significant changes were made to the 2018 FHTC event, which was hosted in early April.

#### Methods

Two public shopping malls were selected as the venues for the 2018 FHTC through community partnerships between the shopping malls and The Corporation. The event ran at both locations concurrently, with one prominent retail mall located in the central/southwest area of Calgary, and the other a popular outlet mall located at Calgary's northern city limit. These locations were selected as trial locations, in attempts to achieve high visibility of the FHTC for Calgary residents and surrounding areas, and are high-visitor volume malls. Unlike previous years (2015–2017), no online pre-registration was offered in 2018. Rather, interested attendees were able to stop by cordoned areas in the two malls to join in the health event. Given the novelty of hosting concurrent events in public spaces, the 2018 event was limited to adults ≥18 years of age. As with all previous FHTC events, the 2018 FHTC event was promoted via all of The Corporation teams' social media, at games, on the websites, by direct email to season-ticket holders, and on Calgary television and radio.

The 2018 FHTC focused mainly on health screening with the continuation of one-on-one health assessments done by nurses/physicians. Representatives from provincial health services were present and actively enrolling at-risk individuals into chronic disease management programs. These programs come at no expense to patients and are hosted by the provincial health authority for those at highest risk of cardiovascular disease, type II diabetes, and other chronic, lifestyle-affected disease (35). Finally, the YMCA hosted booths offering information about memberships at local recreation facilities including financially-supported memberships, as well as free memberships to children currently enrolled in sixth grade, a long standing initiative offered jointly by the Calgary Flames Foundation for Life, The Corporation's charitable branch, and YMCA Calgary.

#### Results

The two mall locations saw differing numbers of walk-up participants. At the central/southwest Calgary mall location, a total of 76 individuals were hosted at the health screening stations; 48 (63%) had elevated CanRisk scores indicating that they would benefit from a diabetes screening test conducted by their primary care physicians. Among the 76 individuals, a total of 14 (18%) had BP readings of ≥140/90 indicating an elevated BP (34), and 10 (13%) did not have a family physician and were thus referred to the College of Physicians and Surgeons of Alberta (CPSA) listing of physicians accepting new patients. At the second mall located just north of the Calgary city limits, a total of 31 individuals were seen at the screening stations, among whom 21 (68%) had elevated CanRisk scores. Of the 31 people assessed, 5 (16%) had elevated blood pressure of ≥140/90, and 1 (3%) did not have a family physician and was thus referred to the CPSA listing.

## Discussion

Professional sporting teams have the ability to leverage their brand and increase awareness around a number of causes including health promotion and healthy lifestyles. The primary aim of the FHTC annual event has been to increase awareness of modifiable health behaviors such as PA and nutrition, access to recreation in local communities, and for participants to receive individualized health screenings with local physicians and nurses. By making use of The Corporation's brands, the permanent presence of these sporting clubs in Calgary may be able to encourage and inspire community-wide health behaviors. Unlike short-term large-scale sporting events such as the Olympic Games and FIFA World Cup, the consistent presence of local professional sports teams may be well-suited to long-term community health promotion though further investigation is needed.

Funding for health initiatives is a key issue for health promoters (4, 8). However, with the financial support and contribution of local sporting franchises, this concern may be overcome. Sporting organizations are poised to develop and disseminate initiatives that increase healthy behaviors through the use of high profile teams, players, within stadia, and through media and community partnerships (7, 8). Through mutually beneficial partnerships, professional sporting organizations have the opportunity to raise their philanthropic and community outreach profiles by providing an arena for health professionals to promote initiatives within the community. Long-term partnerships between these agencies may mitigate concerns of program sustainability, as might targeting third party partnerships with local health promotion agencies, amateur sporting clubs, and youth engagement organizations.

### Lessons Learned

Attendance rates from the four FHTC events suggest there is community interest in health promotion events hosted by local professional sporting teams. While it is likely that use of The Corporation's brand increased awareness of the event, it is unclear whether this was a driving factor for event attendance. However, event promotion was limited to several weeks prior to event delivery; a more sustained approach to event promotion over several months may have increased awareness and potential attendance among the Calgary public. For those attending, the variety of stations and health topics offered at the events from 2015–2017 may have contributed to interest in the event, including the opportunity to meet high-profile Olympic and professional athletes. However, regional data suggest that 69% of individuals living in Calgary self-report personal health as good or excellent (36). It remains unclear then, if participants attended the events as a means to improve poor health, or if attendance was primarily among individuals that were already largely in good health, and were simply attending for personal entertainment. Similar to previous findings, attendees appear to be already-active individuals, interested and motivated to improve health outcomes (22). It is possible that the large-scale, multi-activity iterations of the FHTC events were more appealing to already-healthy, already-active individuals rather than serve to identify at-risk individuals. Given that many survey respondents cited attending primarily for health improvement education, and that a majority of respondents also reported an intention to modify an unhealthy behavior, it is likely that the FHTC events were impactful for increasing health behavior awareness.

Findings from the 2017 FHTC survey though limited, supported recommendations made by the steering committee for event offerings, and the opportunity of the FHTC to serve as a health promotion event. Low response rates to the 2017 survey point to insufficient participant recruitment approaches which may have been attributed to the large-scale nature of the event; individuals were dispersed throughout the WinSport campus while data collection teams remained in one static location near the entry location and electronic surveys were not available for completion at a later date. Nonetheless, 2017 survey results provided guidance for the 2018 event, which focused primarily on health screening and education. Changes made to the 2018 event included offering only walk-up as opposed to pre-registered health screening with nurse/physician teams, which did not contribute significantly to variance in health screens performed. Even so, each iteration of the FHTC provided the Calgary community with an opportunity to engage in learning about or further improving personal health though it has yet to secure known behavior change.

### Future Directions

Collaborative approaches between health promotion professionals and professional sporting teams need to consider the balance between health promoting initiatives in alignment with the community, as well as the sporting team's brand and its fan base. Previous work suggests that partnerships between sporting franchises and public health may be guided by diverging interests (7). In this regard, we anticipate that the descriptions of four annual FHTC events will be of interest to other professional organizations considering such events.

The FHTC events were a collaboration between The Corporation and public health in the sense that FHTC events were guided equally by public health approaches to health promotion, and franchisee knowledge and ability to execute large-scale offerings. This contrasts previous work published in this area that appears to have been research-driven intervention approaches that functioned within regular franchise operations (4, 8). However, large-scale, community based offerings make formal evaluation difficult for researchers hoping to improve the health of their communities. Future work in this area may wish to focus on collaborative approaches that involve more targeted evaluation methods including post-event follow-up, to determine the efficacy, reach, and impact of such events. Further, public health professionals working in collaboration with professional franchises may wish to establish more equal partnerships in terms of event management and community partnerships. This may ensure that logistics such as event location and promotion are considered by all contributing parties, to fully leverage the sporting organization's brand in tandem with public health messages. In this vein, event organizers benefitted from knowing potential attendance rates via free, online event registration, it may be an unnecessary process for future events focused on health screening or identification of at-risk individuals. Yet the events in 2015–2017 generally attracted already active individuals, which may have been the result of association with The Corporation's brand as a sporting franchise collective; professional athletes are often viewed synonymously with healthy behaviors such as exercise and quality nutrition (7). Community-based approaches such as was offered in 2018 may be more effective at attracting at-risk groups. Finally, future research may wish to incorporate multi-evaluation models to determine overall program impact, employing a true community-based participation research model. Public health researchers should assess event feasibility as well as short and long term impacts to determine suitability for all contributing parties to large-scale health promoting events that are offered in collaboration with local professional sporting organizations.

Although it has been over a decade since the San Francisco Padres and other Major League Baseball teams partnered on public health initiatives such as in-stadium PA breaks and nutritious foods at concession stands (7), the literature examining joint efforts between professional sports teams and public health remains sparse. It may be that professional sporting organizations are guided by differing expectations than health promoters, and that the public opinion of the event regardless of its success, falls squarely on the brand of the involved team (7). This may leave professional teams interested in performing in-house evaluations of health promotion initiatives with a brand-endorsing lens. Nonetheless, health promotion events such as these including the FHTC would benefit from regular, systematic evaluations to inform future projects. Public health experts and agencies eager to partner with professional sporting organizations may wish to build-in more rigorous evaluations for such events as joint outcomes in and of themselves.

Overall, this review provided support for the collaboration of public health professionals and local professional sporting teams to promote healthy lifestyle behaviors. Given the permanent presence of such teams, there is potential for these organizations to augment public health initiatives by leveraging their brands in promoting health messaging and eliciting positive intentions to change, across a variety of demographics, socioeconomic backgrounds, and the local population as a whole.

## Data Availability Statement

The raw data supporting the conclusions of this article will be made available by the authors, without undue reservation upon reasonable request.

## Ethics Statement

The studies involving human participants were reviewed and approved; ethical approval was obtained by the University of Calgary Cojoint Health Research Ethics Board, Ethics ID: REB17-0399. Participants provided informed, written consent to participate.

## Author Contributions

EO and WG headed the data collection and analysis. EO wrote the first draft of the manuscript. EO, WG, TB, and GM contributed to survey development. EO, WG, KB, and GL were members of the Flames Health Training Camp Event Advisory Group and contributed to intervention and event planning. All authors provided input into the final version of this manuscript.

## Conflict of Interest

KB was employed by the company Innovative Sport Medicine and GL was employed by the company Canadian Pacific Railway. The remaining authors declare that the research was conducted in the absence of any commercial or financial relationships that could be construed as a potential conflict of interest.
